# Functional Connectivity Changes in Resting-State EEG as Potential Biomarker for Amyotrophic Lateral Sclerosis

**DOI:** 10.1371/journal.pone.0128682

**Published:** 2015-06-19

**Authors:** Parameswaran Mahadeva Iyer, Catriona Egan, Marta Pinto-Grau, Tom Burke, Marwa Elamin, Bahman Nasseroleslami, Niall Pender, Edmund C. Lalor, Orla Hardiman

**Affiliations:** 1 School of Medicine, Trinity College Dublin, Dublin 2, Ireland; 2 School of Engineering, Trinity College Dublin, Dublin 2, Ireland; 3 Trinity Centre for Bioengineering, Trinity College Dublin, Dublin 2, Ireland; 4 Academic Unit of Neurology, Trinity Biomedical Sciences Institute, Trinity College Dublin, Dublin 2, Ireland; Children's Hospital of Pittsburgh, University of Pittsburgh Medical Center, UNITED STATES

## Abstract

**Background:**

Amyotrophic Lateral Sclerosis (ALS) is heterogeneous and overlaps with frontotemporal dementia. Spectral EEG can predict damage in structural and functional networks in frontotemporal dementia but has never been applied to ALS.

**Methods:**

18 incident ALS patients with normal cognition and 17 age matched controls underwent 128 channel EEG and neuropsychology assessment. The EEG data was analyzed using FieldTrip software in MATLAB to calculate simple connectivity measures and scalp network measures. sLORETA was used in nodal analysis for source localization and same methods were applied as above to calculate nodal network measures. Graph theory measures were used to assess network integrity.

**Results:**

Cross spectral density in alpha band was higher in patients. In ALS patients, increased degree values of the network nodes was noted in the central and frontal regions in the theta band across seven of the different connectivity maps (p<0.0005). Among patients, clustering coefficient in alpha and gamma bands was increased in all regions of the scalp and connectivity were significantly increased (p=0.02). Nodal network showed increased assortativity in alpha band in the patients group. The Clustering Coefficient in Partial Directed Connectivity (PDC) showed significantly higher values for patients in alpha, beta, gamma, theta and delta frequencies (p=0.05).

**Discussion:**

There is increased connectivity in the fronto-central regions of the scalp and areas corresponding to Salience and Default Mode network in ALS, suggesting a pathologic disruption of neuronal networking in early disease states. Spectral EEG has potential utility as a biomarker in ALS.

## Introduction

Amyotrophic Lateral Sclerosis (ALS) is an age-related neurodegenerative disorder of relentless progression and fatal outcome. It has a lifetime risk of 1:300 [1]. There is compelling evidence that ALS affects domains outside of the motor system, including cognition. Thirteen percent of those with ALS exhibit a full blown frontotemporal dementia (FTD), and a further 40% have evidence of progressive cognitive and behavioral impairment [2, 3]. There is also considerable heterogeneity in genotype. Up to 11% of those with ALS carry a hexanucleotide repeat expansion on Chromosome 9p21 and these individuals are more likely to exhibit deficits in cognition and behavior [4]. Up to 40% of those with ALS remain cognitively unaffected and experience a slower disease trajectory compared to those with executive impairment [2].

There is an urgent need for clinically useful biomarkers of diagnosis and progression in ALS. While neurophysiologic measurement tools including electromyography (EMG)-based Motor Unit Number Estimation (MUNE) and Motor Unit Number Index (MUNIX) can measure progressive loss of motor units and are useful and reliable predictors of clinical progression [5], reliable markers of upper motor neuron dysfunction remain elusive.

ALS necessarily leads to alterations in motor networks of the brain. Measurement of neural connectivity therefore represents a potentially useful clinical biomarker that encompasses both motor and cognitive decline.

Connectivity measurements can be structural, functional and effective [6]. Structural connectivity explores the anatomic connections between different brain areas; whereas functional connectivity refers to an inferred relationship between brain regions based on statistically similar patterns of activation over time. Finally, effective connectivity measurements not only infer statistical relationships between regions, but also make inferences as to how activity in one brain region influences another region, either in an excitatory or inhibitory manner [7].

Analysis of resting-state functional connectivity using both model-based and model-free approaches, has suggested the existence of at least three major networks: (*i*) a central executive network (CEN), the key nodes of which include the dorsolateral prefrontal cortex (DLPFC), and posterior parietal cortex (PPC); (*ii*) the default-mode network (DMN), which includes the ventro-medial prefrontal cortex (VMPFC) and posterior cingulate cortex (PCC); and (*iii*) a salience network (SN), which includes the ventrolateral prefrontal cortex (VLPFC) and anterior insula (jointly referred to as the fronto-insular cortex; FIC) and the anterior cingulate cortex (ACC). During the performance of cognitively demanding tasks, the CEN and SN typically show increases in activation whereas the DMN shows decreased activation [8].

Recent studies of functional connectivity in ALS using Magnetic Resonance Imaging (MRI) have suggested altered network integrity in salience and default mode networks [9, 10]. However, MRI is costly and many patients cannot cooperate, particularly in those with respiratory failure, and in those with worsening cognitive and behavioral impairment. The lack of transferability of findings across different scanners is also an important limitation. Moreover, signal measured by MRI represents hemodynamic changes in the brain which serve only as an indirect measure of neural activity.

Advanced neurophysiologic measurements including magnetoencephalography (MEG) and high resolution electroencephalography (EEG) have a number of advantages compared to MRI. While MEG requires specialized equipment housed in a dedicated setting, high density surface EEG recording is relatively inexpensive, widely available and easily applied in a clinical setting [11]. EEG data are more closely linked to real-time neural activity with a much greater temporal resolution (milliseconds) than the hemodynamic MR signal (seconds). This is particularly important in measuring connectivity based on statistical relationships. The improved temporal resolution of EEG allows specific analysis of such relationships within a number of well-known frequency bands, each of which has characteristic biological and pathophysiological significance. This partitioning of the time-varying EEG into different frequency bands is commonly known as spectral EEG and is typically performed using the Fourier transform [12]. The output of this transform is a number of numerical coefficients each of which indexes the power in the EEG signal at a particular frequency. Variations in these values can then be examined over time and compared between different brain areas to obtain indices of functional and effective connectivity [11, 13].

Functional connectivity is assessed by examining statistical relationships between activity in different brain regions (or data from different electrodes): These can be directed and undirected measures. Undirected measures assess simultaneous activation of regions, while directed measures attempt to take into account directionality of connections (to determine whether the activity of one region leads or lags activity in the other region). The directed approach can be more useful as it provides measures of both forward and backward connectivity between regions [7].

Once functional connectivity between different brain regions has been estimated, the connection strengths (or “weights”) can then be represented as a connectivity map. In mathematics such a map is known as a graph and it can be quantified using mathematical graph theory [14]. Studies in Alzheimer’s disease using quantitative graph theory measures suggest that commonly observed patterns of network activity disintegrate to a random pattern as the disease progresses [13]. Conversely, the limited network studies that have been performed in FTD suggest an evolution towards a more ordered network structure, possibly reflecting a different underlying patho-physiological process [13]. In particular it has been postulated that the salience network becomes more structured as dementia progresses [13]. Activity in these networks is thought to control switching between resting state behavior and active task engagement.

As there is evolving evidence of overlap between ALS and frontotemporal impairment, with up to 13% of ALS patients fulfilling the clinical criteria for FTD [3], the aim of the present study was to explore functional connectivity in a cohort of ALS patients with particular reference to the underlying salience network, and to evaluate the possible utility of spectral EEG-based network measures as disease biomarkers.

## Materials and Methods

### Subject demographics

A total of 18 patients with non-familial ALS (6 female; mean age 56 years, range 42–67 years) and 17 healthy controls (7 female; mean age 51 years, range 30–78 years) were recruited after obtaining informed written consent. 15 Patients had spinal onset, 2 respiratory onset and 1 bulbar onset disease. All patients were of Irish nationality. All patients were within 18 months of disease onset, all fulfilled the El Escorial diagnostic criteria for Possible, Probable or Definite ALS, and all had undergone detailed neuropsychological testing using an established neuropsychology battery of tests (Table C in S1 File) [3] and did not exhibit any evidence of cognitive or behavioral impairment. ALSFRS-R was performed at the time of recording. All were tested for known genes including hexanucleotide repeat expansions in C9orf72 and were negative.

All patients were on Riluzole 50 milligrams twice daily. This was not stopped or changed during the study. They were not on any other medications. All the patients were tested earlier for respiratory insufficiency and were found to be normal, and none of the tested patients were on non-invasive ventilatory support. None of the patients suffered from mood disorders or sleep disturbances.

The research was approved by the Ethics Committee of Beaumont Hospital.

### Data acquisition

EEG data were recorded in a dedicated laboratory from 128 scalp electrode positions, filtered over the range 0–134 Hz and digitized at 512 Hz using an ActiveTwo system (BioSemi B.V, Amsterdam, Netherlands). Each subject was fitted with an appropriately sized EEG cap.

Resting state EEG was recorded from all subjects. Each subject was asked to relax while fixating on an ‘X’ on a sheet of paper in order to reduce eye movement artifacts. Three 2-minute blocks of data were recorded while subjects had their eyes open and one block of two minutes with eyes closed. The sampling rate of the EEG was 512Hz which facilitated avoidance of high frequency aliasing when analyzing frequencies up to 100Hz.

### Data preprocessing

Preprocessing of the raw EEG data was performed with an EEG processing toolbox (FASTER) [15], based within the MATLAB software environment (Mathworks, Natick, MA, USA).

Segments of 128 channel EEG data uncontaminated by eye-blinks or muscle artifacts were visually selected from the preprocessed data. These were subsequently divided into 2 second epochs to facilitate subsequent analysis.

The preprocessing included statistical identification of bad channels (i.e. those contaminated by noise), and the replacement of data on those channels by a weighted average of the data from neighboring clean channels. Low pass (100 Hz), high pass (0.5 Hz) and notch (50 Hz) filters were applied to the digitized signals to remove high frequency noise, noise due to baseline drift, and noise from the electrical mains supply, respectively.

### Data analysis

**Fig 1:**
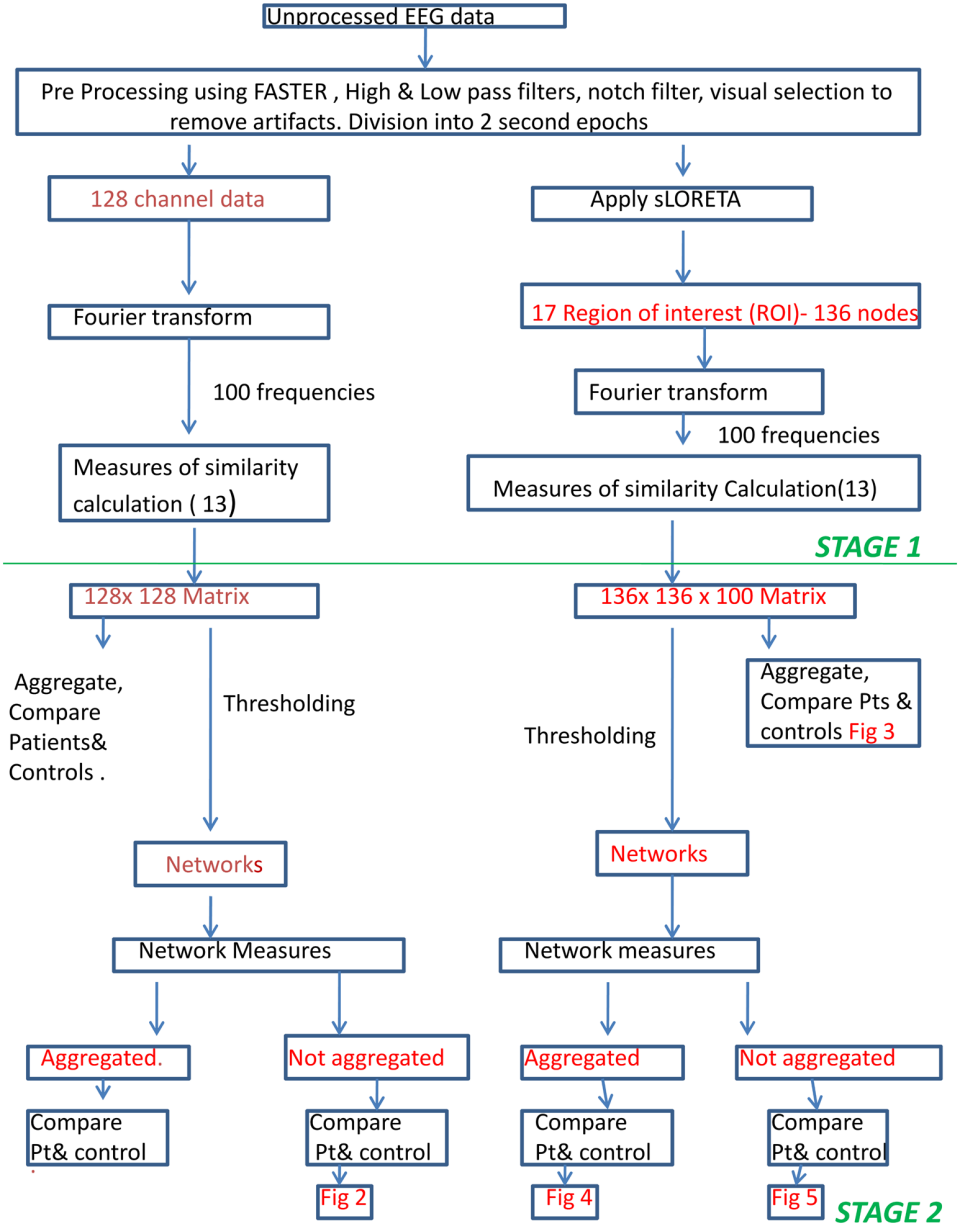
Methods Flow Chart. Flowchart of methods explaining the preprocessing and two stage processing for scalp connectivity and nodal connectivity.

Data analysis was undertaken in two modes, scalp measure-based connectivity and nodal connectivity. The earlier refers to analyzing connectivity measures based on the data recorded directly from the scalp electrodes while the latter is based on the activity of the anatomically localized sources of those scalp data (Fig 1).

#### Data analysis—scalp based connectivity

Data analysis was undertaken in two stages. The first stage involved calculating various quantitative measures of similarity between the EEG data on each pair of 128 electrodes for each frequency band of interest. This produced indices of the interdependent connectivity between each pair of electrodes in the form of 128 x 128 matrices, one matrix for each quantitative measure used and for each frequency band. The second stage then involved an analysis to determine which electrode pairs showed connectivity strengths that were statistically greater than chance. This resulted in a graph of connections for each subject for each quantitative measure (Fig 1). These graphs were then quantified using mathematical graph theory [16]. Our analysis then involved searching for statistical differences in these graph theoretic measurements between patients and controls.

*Stage 1*: Frequency analysis was performed for each subject by calculating the fast Fourier transform (FFT) of all their selected epochs. This was performed at 100 frequencies evenly spaced between 1Hz and 100Hz. Various connectivity measures (Table A in S1 File) are calculated on the frequency domain representation of the data. An exception was the calculation of measures based on auto-regressive (AR) modelling of EEG time series (i.e. Granger Causality, Directed Transfer Function and Partial Directed Coherence) where spectral analysis is performed after the AR modelling.

*Stage 2*: Once the 128 x 128 matrices of similarity were computed, we then conducted an analysis to determine which electrode pairs showed connectivity strengths that were statistically greater than chance. This was undertaken within each of the following characteristic EEG bands: delta, theta, alpha, low beta, high beta and gamma frequency bands (1–3Hz, 4–7Hz, 8–13Hz, 14–21Hz, 22–30Hz and 31–60Hz, respectively). Similarity measures at different individual frequencies were averaged together to form aggregate measures for each frequency band.

We then performed two-sample unpaired student t-tests on each of the six frequency bands: delta, theta, alpha, low beta, high beta and gamma to compare between controls and subjects. Due to the large number of interconnected electrode pairs (8128 pairs for undirected measures) statistical significance was calculated to satisfy a corrected significance level (p<0.0005) [17] to account for effects of multiple testing. All electrode pairs that showed significant relationships using this criterion were deemed to be connections in an overall network of neural function, allowing generation of a graphical representation of connectivity differences between groups.

For graph-theoretic analysis, matrices were thresholded to include only the top 20% results as an initial way of filtering the results to include most significant results. Subsequently, the network connectivity measures of the resultant graphs were calculated. ((Table B in S1 File) Quantitative comparison of graph theory-based measures of network connectivity differences between groups required a different approach: the similarity measures were not averaged across frequencies in the first place. Connectivity measures were assessed at each individual frequency between 1 and 100 Hz permitting identification of “clusters” of statistically significant differences between patients and controls across both electrode and frequency.

The connectivity measure of assortativity produces one measure for the entire network, as opposed to a measure for each electrode. Accordingly, we averaged the assortativity measure across frequencies to obtain aggregate measures for each of our six frequency bands as before.

Network measures were calculated using FieldTrip toolbox in MATLAB [18].

#### Data analysis–nodal analysis

We assessed connectivity between brain regions that were likely contributing to scalp recordings. To do this we followed the same two stage process on the data as outlined above, after performing source analysis to estimate which brain regions were contributing to our EEG signal. This source localization was done using sLORETA software [19] that uses the Montreal Neurological Institute (MNI) coordinate system [20]. In this study, we specified 17 brain regions of interest (ROI) out of 136 nodes in source analysis. These 17 ROIs (named in supplementary material) correspond to the Default Mode Network (DMN), Central Executive Network (CEN) and Salience Network (SN).

The output signals of sLORETA were analyzed in the same way as the electrode-level data mentioned above. The connectivity between ROIs was calculated, using the same two analysis stages, for all of the same connectivity measures described above.

Likewise, the same method for statistically comparing the connectivity between controls and patients was used with an alpha value of p = 0.05 for nodal network analysis.

### Statistical analysis

#### Scalp connectivity/nodal connectivity

For scalp connectivity, p value was set at p<0.0005 based on similar EEG studies in degenerative conditions which were specifically looking for p value levels to account for multiple testing [17].

#### Scalp network connectivity/nodal network connectivity

For scalp network analysis, a 2 sample unpaired t-test with alpha value of 0.02 was used. For nodal network analysis, a 2 sample unpaired t-test with alpha value of 0.05 was used. We applied more stringent criteria to the scalp network analysis to account for larger number of connection pairs in scalp network compared to nodal network (8128 vs. 136).

#### Test for discriminatory power

We further analyzed the measures with the highest significant between-group differences to assess the potential for discrimination for use in clinical screening or confirmatory diagnosis. This included comparing the distribution of the measure for patients vs. controls, as well as calculating the receiver operating characteristic (ROC) curve [21]. The ROC curve is used to find the best combination of specificity and sensitivity of the optimal binary classifier based on this measure. Calculations were performed in MATLAB.

## Results

Connectivity measures were assessed in four levels namely scalp connectivity, scalp network connectivity, nodal connectivity and nodal network connectivity. Scalp connectivity and nodal connectivity refers to results calculated without thresholding, while scalp network connectivity and nodal network connectivity are calculated after thresholding (Fig 1).

### Scalp connectivity

Cross Spectral Density showed significant differences between patients and controls. This occurred for data in the alpha band and theta band frequency ranges over parietal scalp, with higher values for patients (p<0.0005). No significant differences were noted using other measures of scalp connectivity in any frequency band.

### Scalp network connectivity

**Fig 2:**
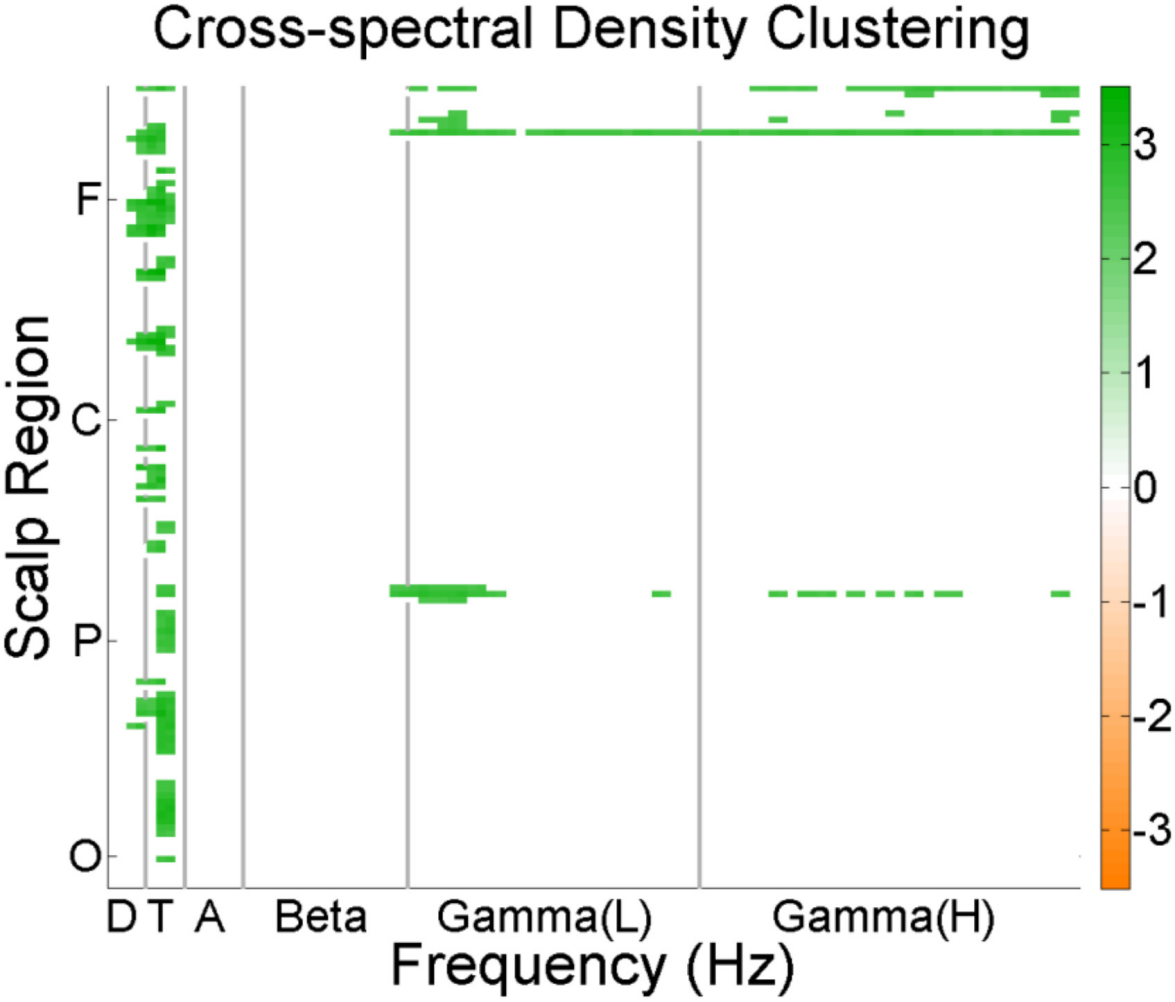
Scalp network connectivity. Shows difference in clustering coefficients between patients (green) and controls (orange) in different scalp regions. (F = Frontal, C = Central, P = Parietal O = Occipital) (D = Delta, T = Theta, A = Alpha L = Low, H = High).

Among patients, increased Degree values of network nodes were recorded in the central and frontal regions in the theta band across seven different connectivity maps (coherence, cross spectral density, phase locking value, partial directed coherence, power correlation, pairwise phase consistency and weighted pairwise phase consistency). The Clustering Coefficient was increased in the theta range in ALS patients compared to controls across all regions of the scalp. Gamma range Clustering Coefficient was also increased in the occipital, parietal and frontal regions in the ALS patients compared to controls. This was observed for all of the following connectivity maps: coherence, CSD, phase locking value, partial directed coherence, power correlation, pairwise phase consistency and weighted pairwise phase consistency (Fig 2).

Using ratio of variances (or F-ratio), which undertakes comparisons using an F-distribution, higher connectivity was noted in the patient group compared with the control group, however these differences were not statistically significant.

### Nodal connectivity

**Fig 3:**
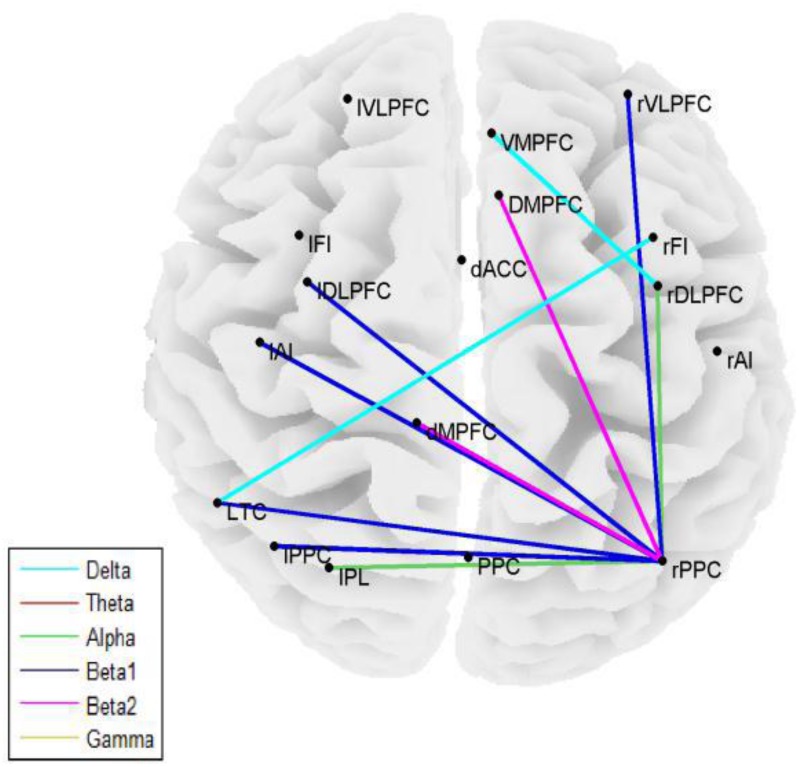
Nodal connectivity. Statistically significant differences in nodal connectivity (Directed Transfer Function) between patients and controls in various frequency bands.

Statistically significant differences between ALS patients and controls (p = 0.01) were identified in four of the 12 methods used to calculate connectivity, namely Partial Directed Coherence, Directed Transfer function, Granger Causality, and Weighted Phase Lag Index (Fig 3). This included all of the undirected or effective measures in addition to weighted phase lag index.

Additionally, correlation based connectivity measures, (Coherence, Power Correlation, Phase locking value, pairwise phase consistency) differed significantly between patients and controls (p = 0.05). Specifically, differences in alpha band directed transfer function were statistically significant in anterior insular cortex, inferior parietal cortex, and ROI from frontal regions (p = 0.01).

### Nodal network connectivity

**Fig 4:**
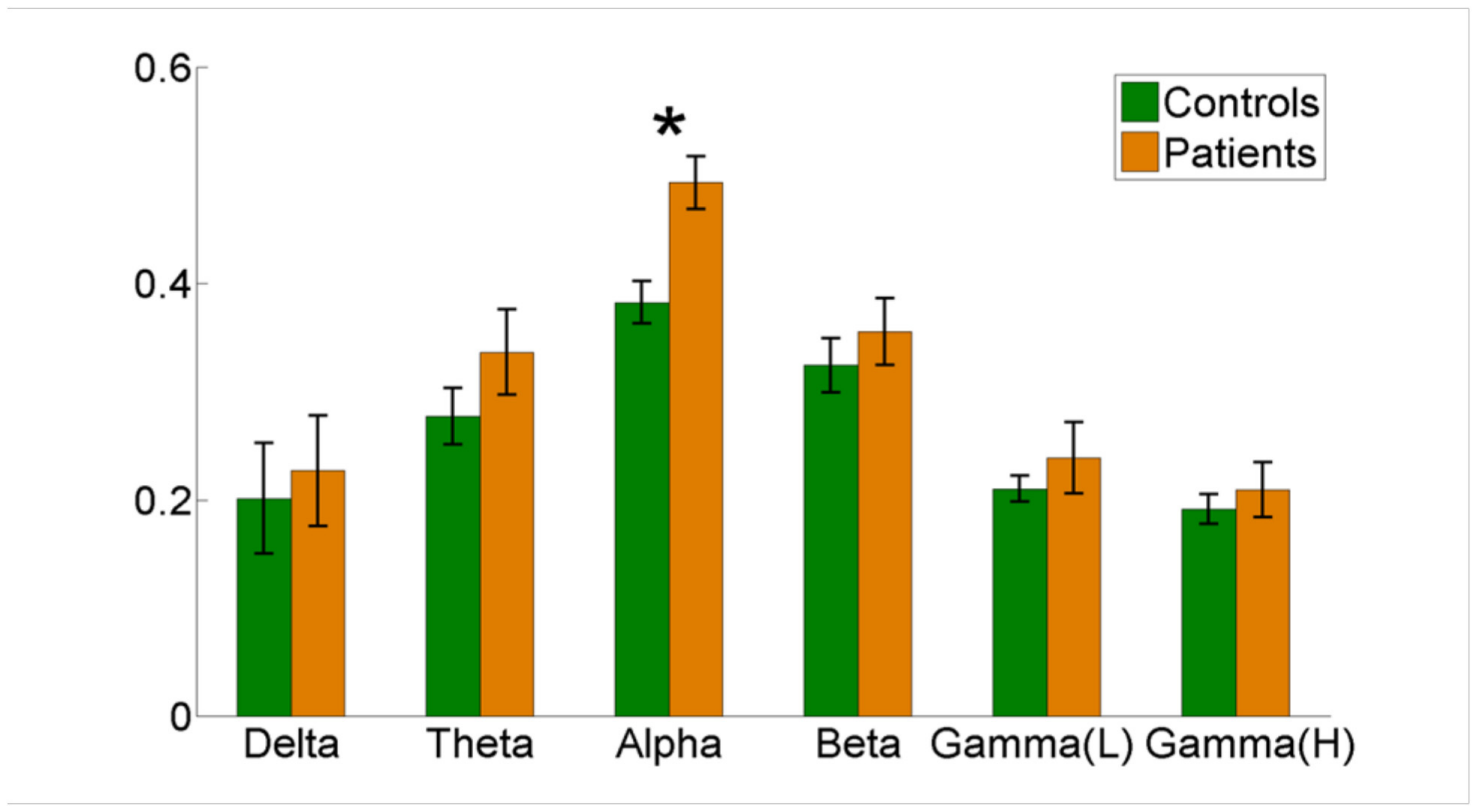
Aggregated nodal network connectivity. Assortativity of Partial Directed Coherence, showing differences between patients (Yellow) and controls (Green) with p = 0.0032 for Alpha range (Delta p = 0.74, theta p = 0.26, beta p = 0.46, gamma p = 0.47).

**Fig 5:**
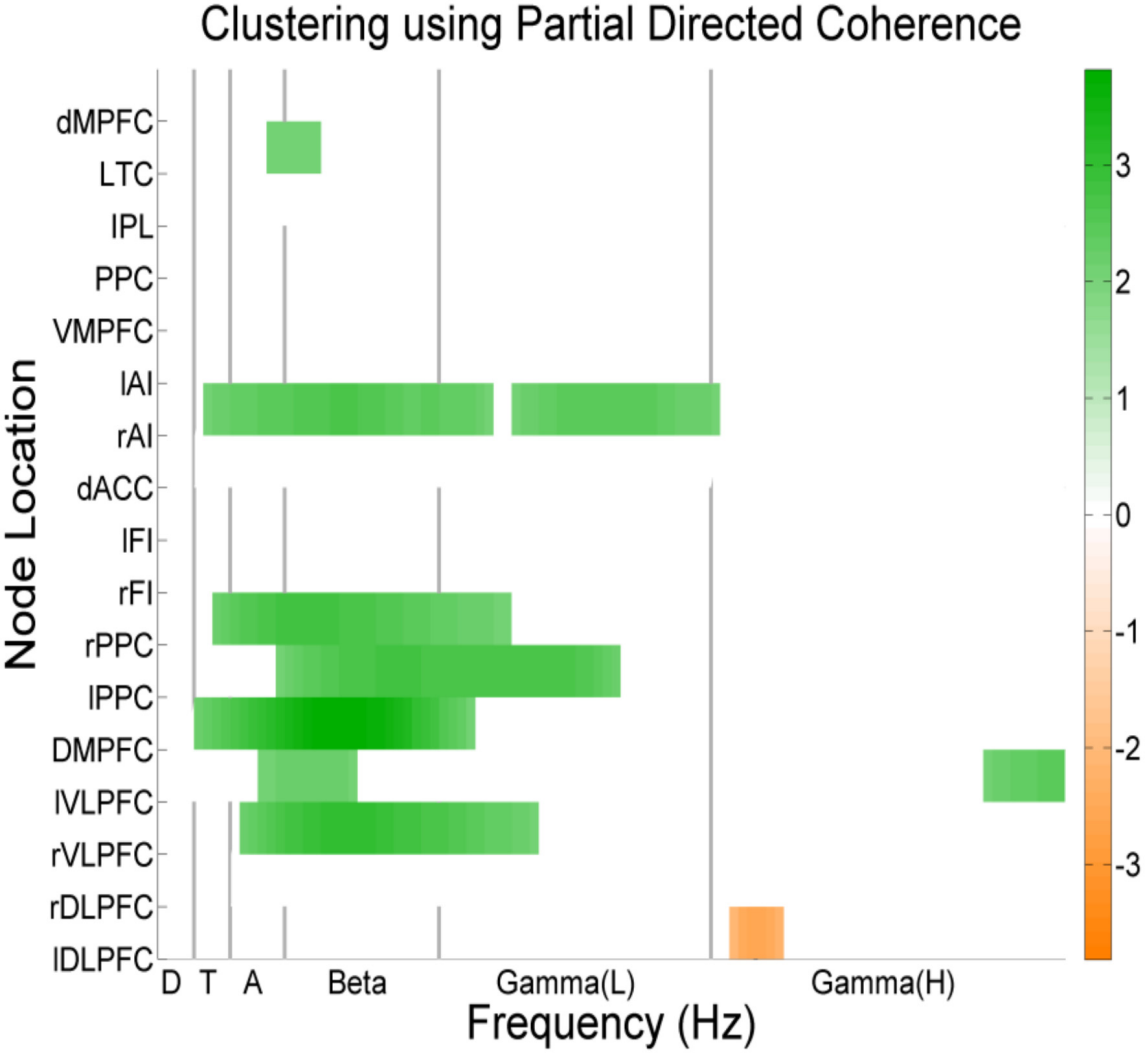
Non-aggregated nodal network connectivity. Clustering coefficient of PDC values in nodal analysis for beta band for patients (green) and controls (orange).

Significant differences between the patients and controls were present in assortativity of PDC connectivity in the alpha band (p = 0.0032) (Fig 4). Additionally, statistically significant increases were present in the Degree of PDC and Coherence values in the Anterior Cingulate Cortex and Fronto-insular Cortex in beta and gamma frequencies (Fig 5).

Significantly higher values for Clustering Coefficient in PDC was identified for patients in Anterior Insular Cortex, Dorsomedial and Ventrolateral Prefrontal cortex, Posterior Parietal Cortex across alpha, beta, gamma, theta and delta frequencies.

### Discriminatory power

**Fig 6:**
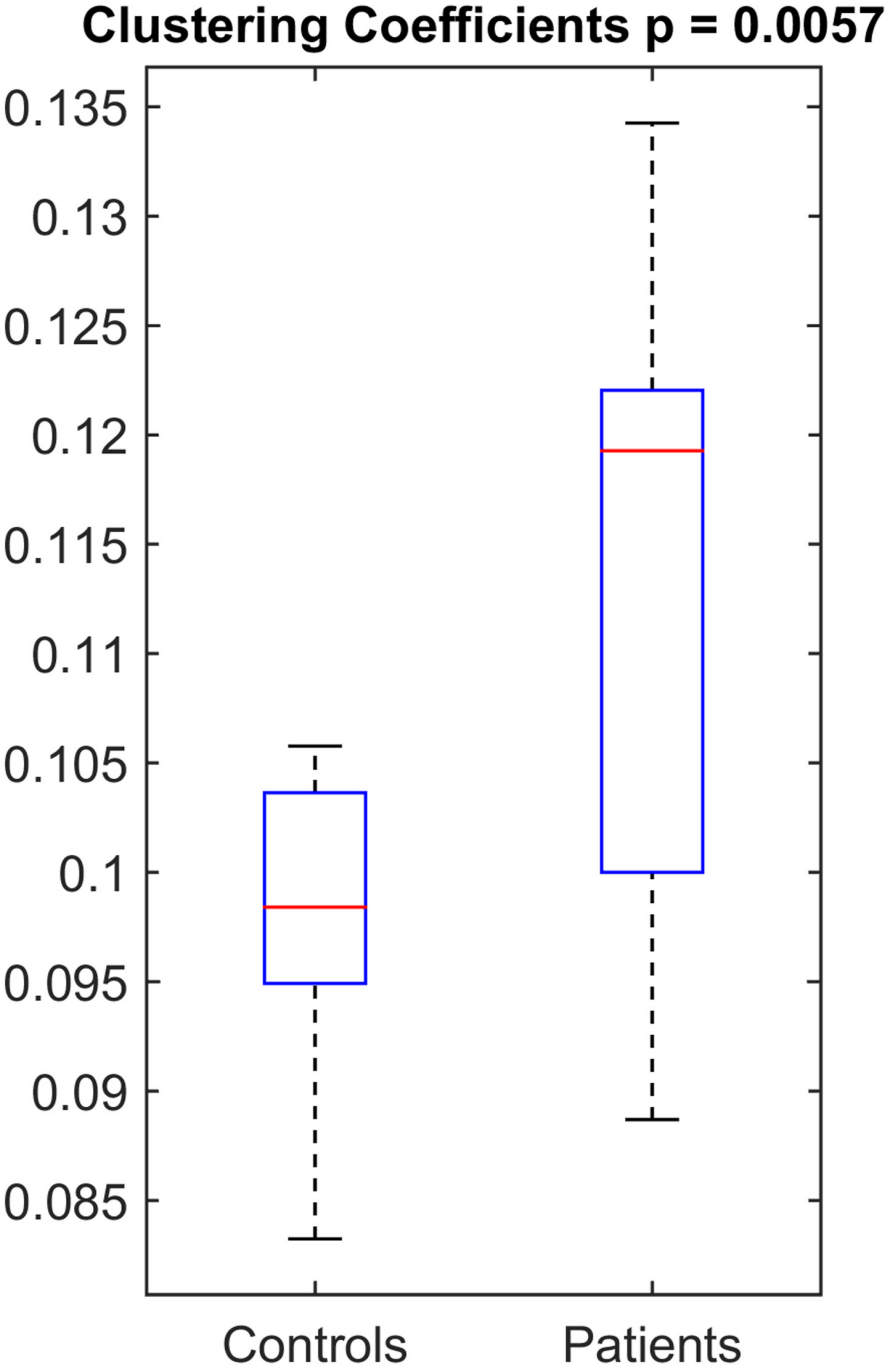
The distribution of clustering coefficient of PDC in beta band as a box-plot in patients vs. controls, showing the median and interquartile range.

The measure with the most significant and most consistent (across various bands and various regions) between-group difference, i.e. the clustering coefficient of PDC values in nodal analysis for beta band, was tested for the level of afforded discrimination. Fig 6 shows the median, interquartile range and range of this measure in boxplots for patients vs. controls. The median clustering coefficient of PDC in beta range was 0.12 for patients while it was 0.098 for controls. The p-value of the distributions was 0.0057. The ROC analysis to determine the sensitivity and specificity of this measure showed that the optimum-threshold classifier has a sensitivity of 58% and specificity of 100%.

## Discussion

We have shown that spectral EEG measurements in ALS patients are different from healthy age-matched controls. All ALS patients had undergone detailed neuropsychological testing and, as a group, did not differ from controls. Scalp nodal connectivity was consistently higher across 7 connectivity measures tested in fronto-central region, showing an increased number of connections in network nodes in these regions in ALS patients compared to controls. Similarly, clustering coefficient, which measures the triangular connections in a network, and a measure of network efficiency, was increased in ALS patients compared to controls. This measure was consistently increased when tested across various connectivity measures, and is thus robust. As in the case of Degree measurements, these changes occurred predominantly in frontal regions.

Assortativity, which measures the hierarchy of brain networks, was significantly higher among ALS patients compared to controls, reflecting pathologically organized networks [7]. Nodal connectivity measures also demonstrated increased connectivity in the ALS group. Increased connectivity has also been demonstrated in FTD patients [13]. These observations in ALS are congruent with fMRI changes in ALS [10] showing increased connectivity among ALS patients in Default Mode Network and fronto-parietal regions, attributed to enhanced recruitment of cortical networks as compensation for neuronal loss or due to cortical disinhibition. The findings are also consistent with pathological changes reported in ALS [22], and with the known biological link between ALS and FTD [4]. Directed Transfer of Function (an indicator of directional influence of network nodes in a multivariate environment), demonstrated statistically significant increase in connectivity in the frontal cortex, anterior insular cortex, and parietal cortex. These finding are consistent with the role of the anterior insular cortex as the switching area of Salience network, and the anterior cingulate gyrus as a direct relay node next to anterior insular cortex [8].

The neuropsychology tests were all within the normal range for patients who participated in the study. The individual scores of neuropsychology tests did not show any correlation with the Clustering Coefficient or the Degree of PDC connectivity of the Salience network (Spearman’s rank correlation, p > 0.05, Bonferroni corrections with n = 40).

The clustering coefficient of PDC in the nodal network analysis was selected for discriminatory power analysis as the results were consistently spread over multiple adjacent frequency bands and multiple regions with a high p-value which makes the findings unlikely to be due to chance (Type-I error). When we plotted the values separately for patients and controls (Fig 6), their median values were different; however, there is overlap between the distributions of values. Therefore, this measure, though useful on a group basis, might not be immediately applicable on an individual basis. The ROC analysis for this measure showed that the optimum classifier gives a sensitivity of 58% and a specificity of 100%. This again makes this measure useful and important as a confirmatory tool, but probably not yet for screening purposes. Though promising, a study with larger sample size is warranted to assess the feasibility of these measures as a definite biomarker, on an individual basis.

Notwithstanding, taken together, these results suggest increased activity in the salience network in ALS patients. Further evidence of disruption in the salience network included increased nodal network connectivity in insular Cortex, dorsomedial and ventrolateral Prefrontal cortex, and posterior parietal cortex. The involvement of parietal and occipital cortical areas, which form part of default mode network, raises the possibility that hyperactive, pathologically plastic salience network may take over from other networks in the initial stages of disease, leading to the initial formation of giant networks which fail in later stages of disease. The hypothesized giant functional network formation could be a compensation for structural neuronal loss or white matter damage as part of the disease. It could also be hypothesized secondary to pathologic hyper plasticity of the network elements. It could also be secondary to loss of inhibitory control over network regions as part of the disease process.

## Limitations

Although our findings are novel, our study has limitations. The objective was to apply a large group of connectivity measures and various methods to the connectivity and network assessment to a cross section of ALS population that was selected for clinical homogeneity: all patients were within 18 months of first symptom, had undergone extensive neuropsychological assessment and were cognitively intact, were negative for known genetic mutations, and the majority had spinal onset disease (83%). Based on our findings, a more extensive network disruption could be anticipated in ALS patients with associated cognitive and behavioral impairment

Our numbers in this pilot study are small, and the study is cross-sectional in nature and will require replication in larger cohorts with longitudinal follow up. Our focus on this study was to establish the usefulness of high density spectral EEG as a useful tool in bringing out the connectivity changes among ALS patient cohort, with a view to enable expanded studies with larger cohort using multiple modalities. Nevertheless, extension of the study to and validation of the results against other imaging modalities like DTI and fMRI are interesting research directions.

Our data-driven analysis provides credible proof of concept as to the utility of spectral EEG as a novel, inexpensive and clinically applicable biomarker tool of central network disruption in those with ALS and normal cognition. Our data encourages detailed use of longitudinal studies to measure changes in network impairment over time.

## Supporting Information

S1 File Supplementary material on methods. Table A: Explanation of terms used in describing the undirected and directed measures of functional connectivity in this study. Table B: Explanation of terms used in describing network graphs. Table C: Cognitive test battery.
(DOCX)
