# Supplementary material for: Functional Connectivity Changes in Resting-State EEG as Potential Biomarker for Amyotrophic Lateral Sclerosis
Source: PLoS One. 2015 Jun 19;10(6):e0128682. doi: 10.1371/journal.pone.0128682 (PMC4474889; doi:10.1371/journal.pone.0128682)
Supplement: S1 File — Table A: Explanation of terms used in describing the undirected and directed measures of functional connectivity in this study. Table B: Explanation of terms used in describing network graphs. Table C: Cognitive test battery. (DOCX) [file pone.0128682.s001.docx]

Functional connectivity changes in resting-state EEG as potential biomarker for Amyotrophic Lateral Sclerosis

Parameswaran M. Iyer*, ^1,4^, Catriona Egan, ^2, 3^,Marta Pinto-Grau, ^4^,Tom Burke, ^4^, Marwa Elamin, ^1,4^, Bahman Nasseroleslami, ^4^, Niall Pender , ^4^ Edmund C. Lalor, ^2, 3,^ Orla Hardiman,^1,4^

Author Affiliations

^1^ School of Medicine, ^2^ School of Engineering ^3^ Trinity Centre for Bioengineering

^4^ Academic Unit of Neurology, Trinity Biomedical Sciences Institute, Trinity College Dublin, Dublin 2, Ireland.

Corresponding Author

Parameswaran Mahadeva Iyer

Department of Neurology

Trinity Biomedical Science Institute

Dublin 2, Ireland

Email: [parames68@gmail.com](mailto:parames68@gmail.com)

Tel: 00353857444441

**Supplementary materials**

**Methods**

**Scalp based connectivity**

**Stage 1**

In order to compute similarity between the data on pairs of electrodes, we used a MATLAB based toolbox known as Fieldtrip. A number of directed and undirected quantitative measures were calculated. The undirected measures computed were as follows: coherence, amplitude correlation, power correlation, cross-spectral density, phase locking value and pairwise phase consistency. The directed measures calculated were directed transfer function, granger causality, partial directed coherence, phase slope index, weighted phase lag index, debiased weighted phase lag index and weighted pairwise phase consistency. (Table 1E)

**Stage 2**

To compute the assortativity and the degree of the network a binary connectivity matrix was required. This was achieved through thresholding the 128 x 128 matrices. For each frequency band all the connections for both the patients and controls were sorted by value, the value that was 20% from the top was chosen as the threshold. This allowed for 20% of the connections to be present in the binary matrix. Each threshold value was subsequently different for each frequency. To analyze any statistical difference between the ALS patients and the controls a two sample unpaired student t-test with an alpha value of 0.02 for the scalp network.

To test each of the resulting network graphs for significant differences between patients and controls we used a number of mathematical graph theory measures of network connectivity (Table 2E).

FieldTrip[[1](#_ENREF_1)] contains the Brain Connectivity Toolbox (BCT)[[2](#_ENREF_2)] created by Sporns *et al* to calculate the network parameters. Specific network measures calculated included: degree of the network, assortativity, clustering coefficient and the betweenness centrality. We also evaluated networks in brain function as “small world network” as described in graph theory by exhibiting high clustering coefficient and short path length thereby increasing efficiency. The clustering coefficient relates to the level of interconnection in a network. Formally, the clustering coefficient of a node is the number of other nodes connected to it, which also connect to each other, forming a triangle

**Nodal analysis**

sLORETA is a custom program designed for source localization of EEG data; it is the first linear solution to produce zero localization error for test-point sources [[3](#_ENREF_3)]. It uses the recorded EEG and the location of the electrodes to estimate the sources of those signals which can be clustered into a number of nodes of activity. In order to generate the signal for each region of interest (ROI), a radius must be defined so that the signal is localized to a volume instead of a point. The radius used was 15mm, generating nodes with a volume of 14.1x103mm^3^. Total of 17 regions of interest (RO) were used. These 17 regions corresponded to 136 nodes in these specified regions.

The regions included are

| Default mode network  Posterior cingulate cortex |  | |
| --- | --- | --- |
| Ventral anterior cingulate cortex | |  |
| Ventral medial prefrontal cortex | |  |
| Dorsal medial prefrontal cortex | |  |
| Right inferior parietal lobe | |  |
| Left inferior parietal lobe | |  |
| Right inferior temporal cortex | |  |
| Left inferior temporal cortex  Central executive network | |  |
| Left dorsal lateral prefrontal cortex |  | |
| Right dorsal lateral prefrontal cortex | |  |
| Medial prefrontal cortex | |  |
| Right posterior parietal cortex | |  |
| Left posterior parietal cortex  Salience network | |  |
| Right ventral lateral prefrontal cortex |  | |
| Left ventral lateral prefrontal cortex | |  |
| Left& Right frontal insular Cortex | |  |
| Right anterior Cingulate Cortex | |  |
| Left anterior Cingulate Cortex | |  |

Similar to the general scalp analysis, the sLORETA output was initially clustered into frequency groups for general nodal connectivity analysis and when calculating network measures, contribution from each frequency is separately assessed.

To compute the assortativity and the degree of the network a binary connectivity matrix was required. This was achieved through thresholding the matrices, which defines the level below which coupling between voltage derived waveforms does not exist. Statistical difference between the ALS patients and the controls was calculated using a two sample unpaired student t-test with alpha value 0.05 for the ROI network

TABLE A: **Explanation of terms used in describing the undirected and directed measures of functional connectivity used in this study**[[4](#_ENREF_4)].

| **Terms** | **Explanation** |
| --- | --- |
| Correlation | A simple method for comparing the similarity of different signals and can be used to infer the level of functional connection between them. It is measure of how closely two signals correspond and how they vary across time. High correlation is presumed to index a functional connection between two signals. |
| Coherence | Correlation between two time series computed for all frequencies. Depends on phase and amplitude covariance of the signals. |
| Cross spectral density (CSD) | The cross-spectral density is defined as the Fourier transform of the cross-covariance function of the two signals being compared, which may also be described by means of the product of the Fourier coefficients of each of the signals at any one frequency. |
| Phase of a signal | The phase of a wave refers to the ratio of an oscillation cycle (between 0 and 2π) that has been passed from the start of an oscillation cycle at each corresponding frequency. Two waves with the same frequency are "in phase" if they have the same phase and therefore line up everywhere. Waves with the same frequency but different phases are "out of phase."  A “phase shift” represents the amount a second wave has shifted from the original wave. |
| Phase Synchronization | By examining two signals to see if they tend to be consistently in the same phases at the same times, one can derive a definition of their “phase synchronization”. Phase synchronization methods define connectivity based on the phase shift and its consistency between signals. |
| Phase Locking Value (PLV) | The PLV looks at phase synchronization of the signals after Fourier transformation. |
| Phase Lag Index (PLI) | Discards phase variations which can occur due to volume conduction between electrodes or reference electrodes by discarding phase differences close to zero or π. PLI has been used in resting state EEG studies [[5](#_ENREF_5)]. |
| Granger Causality | Method of measuring effective connectivity. It is based on linear auto-regressive models. It tests whether the history of signal helps in forecasting another signal. |
| Directed Transfer of Function (DTF) | Based on linear auto-regressive models and estimates the directional influence of a single pair of channels within an inverse frequency-domain multivariate auto regressive model matrix, derived out of multi-channel EEG recording. |
| Partial Directed Coherence (PDC) | Method again based on (non-inverted) frequency-domain multivariate auto regressive model matrix. The difference between PDC and DTF is that DTF is normalized to the node that receives the signal and PDC is normalized with respect to the sending node. |

TABLE B: **Explanation of terms used in describing network graphs**.[[6](#_ENREF_6)]

| **Terms** | **Explanations** |
| --- | --- |
| Nodes, vertices | Interconnected points of activity (e.g. electrodes or brain regions) |
| Edges | Connections between nodes/vertices. |
| Degree | Number of connections converging on each node. |
| Assortativity | The assortativity coefficient is a correlation coefficient between the degrees of all nodes on two opposite ends of an edge. It is a measure of preference of a node to connect to nodes with similar Degrees. |
| Shortest path length | The shortest distance between 2 different nodes. Shortest path length between two nodes is defined as the smallest number of edges connecting them. |
| Global efficiency | Average inverse shortest path length in a network. |
| Clustering Coefficient | Measure associated with the local efficiency. It calculates the degree of triangulation in a system. Triangulation refers to any two nodes that are connected to a third and each other (forming a triangle) [[7](#_ENREF_7)]. |
| Betweenness Centrality | Describes the ‘hub’ properties of a node, that is, how central it is in the network. The betweenness centrality of a node is the fraction of all shortest paths in the network that contain the given node [[8](#_ENREF_8)]. |
| Random network | A network with a high degree, low clustering coefficient and short average path length. |
| Regular network | Network with high clustering coefficient and a long average path length. |
| Small world network | It has a high clustering coefficient and a short path length. This makes the small-world networks the most efficient for information flow. |

TABLE C **Cognitive test battery [**[**9**](#_ENREF_9)**,** [**10**](#_ENREF_10)**]**

| Executive dysfunction | *Impairment that is two SD below the mean for healthy controls on at least two executive tasks* |
| --- | --- |
| Stroop  Colour- Word Test | (a) Priming trial: patients presented with a multicoloured list of colour names and asked to read as many words as they can in 2 minutes. (b) Inhibitory trial: a similar list is presented but colour names (eg, ‘blue’) are printed in an ink colour not denoted by the name (e.g. red). Correct responses in two minutes are recorded. |
|  | Non-executive factors (including bulbar disability) contribute equally to both trials. The difference in scores between the two trials represent the number of responses ‘lost’ due to the delay imposed by the extra executive demands in the inhibitory trial. |
| Brixton Spatial Anticipation Test |  |
| Backward digit span |  |
| Category fluency | Patients were asked to name as many animals as they could think of in 1 minute (spoken only). |
| Phonemic verbal fluency | Written/spoken, number of words starting with letter ‘S’ generated in 5 minutes and number of four letter words starting with letter ‘C’ generated in 4 minutes. Verbal Fluency Index used to adjust for disability. |
| Memory dysfunction | *Impairment that is two SD below the mean for healthy controls on at least four* *of the parameters highlighted using +* |
| Logical Memory (LM) | +LM1 (immediate recall), +LM2 (delayed recall) and +LM retention (retention) |
| Verbal Paired Associate (VPA) | +VPA1 (immediate recall),+VPA2 (delayed recall) and +VPA retention (retention) |
| Auditory Delayed Recognition Task | +Sum of total recognition scores on Logical Memory and Verbal Paired Associates |
| California Verbal Learning Tests | +Total of five trials, +short delay free recall (immediate recall) +long delay free recall (delayed recall) |
| Rey-Osterrieth Complex Figure test | Non-verbal memory: parameters used: +immediate and +delayed recall trials |
| Language dysfunction | |
| Boston Naming Test | *Impairment that is two SD below the mean for healthy controls on this task.* |
| *Visuo-spatial dysfunction* | |
| Rey-*Osterrieth*Complex Figure Test | *Impairment that is two SD below the mean for healthy controls on copy trial of this task.* |

References

1. Oostenveld R, Fries P, Maris E, Schoffelen JM. FieldTrip: Open source software for advanced analysis of MEG, EEG, and invasive electrophysiological data. Computational intelligence and neuroscience. 2011;2011:156869. Epub 2011/01/22. doi: 10.1155/2011/156869. PubMed PMID: 21253357; PubMed Central PMCID: PMCPmc3021840.

2. Zhou D, Thompson WK, Siegle G. MATLAB toolbox for functional connectivity. NeuroImage. 2009;47(4):1590-607. doi: <http://dx.doi.org/10.1016/j.neuroimage.2009.05.089>.

3. Wagner M, Fuchs M, Kastner J. Evaluation of sLORETA in the presence of noise and multiple sources. Brain topography. 2004;16(4):277-80. Epub 2004/09/24. PubMed PMID: 15379227.

4. Rubinov M, Sporns O. Complex network measures of brain connectivity: uses and interpretations. NeuroImage. 2010;52(3):1059-69. Epub 2009/10/13. doi: 10.1016/j.neuroimage.2009.10.003. PubMed PMID: 19819337.

5. Stam CJ, Nolte G, Daffertshofer A. Phase lag index: assessment of functional connectivity from multi channel EEG and MEG with diminished bias from common sources. Human brain mapping. 2007;28(11):1178-93. Epub 2007/02/03. doi: 10.1002/hbm.20346. PubMed PMID: 17266107.

6. Bullmore E, Sporns O. Complex brain networks: graph theoretical analysis of structural and functional systems. Nature reviews Neuroscience. 2009;10(3):186-98. Epub 2009/02/05. doi: 10.1038/nrn2575. PubMed PMID: 19190637.

7. Watts DJ, Strogatz SH. Collective dynamics of 'small-world' networks. Nature. 1998;393(6684):440-2. Epub 1998/06/12. doi: 10.1038/30918. PubMed PMID: 9623998.

8. Amaral LA, Scala A, Barthelemy M, Stanley HE. Classes of small-world networks. Proceedings of the National Academy of Sciences of the United States of America. 2000;97(21):11149-52. Epub 2000/09/27. doi: 10.1073/pnas.200327197. PubMed PMID: 11005838; PubMed Central PMCID: PMCPmc17168.

9. Byrne S, Elamin M, Bede P, Shatunov A, Walsh C, Corr B, et al. Cognitive and clinical characteristics of patients with amyotrophic lateral sclerosis carrying a C9orf72 repeat expansion: a population-based cohort study. Lancet neurology. 2012;11(3):232-40. Epub 2012/02/07. doi: 10.1016/s1474-4422(12)70014-5. PubMed PMID: 22305801; PubMed Central PMCID: PMCPmc3315021.

10. Phukan J, Elamin M, Bede P, Jordan N, Gallagher L, Byrne S, et al. The syndrome of cognitive impairment in amyotrophic lateral sclerosis: a population-based study. Journal of Neurology, Neurosurgery & Psychiatry. 2012;83(1):102-8.
